# Causal effects of gut microbiota on risk of interstitial cystitis: a two-sample Mendelian randomization study

**DOI:** 10.3389/fmicb.2024.1434117

**Published:** 2024-07-12

**Authors:** Jianguo Gao, Sihai Shao, Yuefan Shen

**Affiliations:** Department of Urology, The First People's Hospital of Huzhou, Huzhou, Zhejiang, China

**Keywords:** interstitial cystitis, gut microbiota, Mendelian randomization, causal relationship, inverse variance weighted

## Abstract

**Background:**

The correlation between gut microbiota and interstitial cystitis has garnered significant attention in previous studies. Nevertheless, the causal relationship between them remains to be clarified.

**Methods:**

Genetic variation serves as a tool in Mendelian randomization analyses, facilitating the inference of causal relationships between exposure factors and disease outcomes. In this study, summary statistics derived from a comprehensive genome-wide association study conducted by the MiBioGen consortium were utilized as exposure factors, while interstitial cystitis data sourced from the GWAS Catalog served as the disease outcome. Then, a two-sample Mendelian randomization analysis was performed by applying inverse variance-weighted, MR-Egger, Weighted Median, Simple Mode, and Weighted Mode. In addition, heterogeneity and horizontal pleiotropy were excluded by sensitivity analysis.

**Results:**

IVW results confirmed that genus *Haemophilus* (OR = 2.20, 95% CI: 1.16–4.15, *p* = 0.015), genus *Butyricimonas* (OR = 2.26, 95% CI: 1.15–4.45, *p* = 0.018), genus *Bacteroides* (OR = 4.27, 95% CI: 1.36–13.4, *p* = 0.013) and *Coprococcus1* (OR = 3.39, 95% CI: 1.28–8.99, *p* = 0.014) had a risk effect on interstitial cystitis. Sensitivity analysis did not find outlier SNPs.

**Conclusion:**

Our analysis has identified a causal relationship between specific genera and interstitial cystitis. However, further validation through randomized controlled trials is essential to substantiate these findings.

## 1 Introduction

Interstitial cystitis (IC) refers to a complex syndrome characterized by persistent pain localized in the urinary bladder, along with symptoms of urinary frequency and/or urgency. This syndrome presents with a wide range of clinical manifestations (Akiyama et al., 2020).

Interstitial cystitis imposes a considerable burden on affected individuals. Recent research on the urinary microbiome has revealed the presence of abundant bacterial populations in urine, suggesting that the composition of these microbiota may be influenced by the menopausal status of the individual and potentially impacting the pathogenesis of IC (Antunes-Lopes et al., 2020).

The influence of gut microbiota composition on the health of distant body organs is well-documented. The pathogenesis of UTIs (urinary tract infections) typically initiates with the periurethral contamination by uropathogens originating from the gut, leading to subsequent colonization of the urethra and ascending migration to the bladder. Uropathogenic *Escherichia coli* (UPEC) constitutes the predominant causative agent, responsible for over 80% of community-acquired UTIs. Given their abundant presence in the gut microbiota of UTI patients, UPEC strains are deemed to originate from the gut (Meštrović et al., 2020).

In conclusion, the gut microbiota may be the ultimate source of bacterial strains responsible for IC. Therefore, exploring the causal relationship between gut microbiota and IC may provide new targets and ideas for the prevention and treatment of IC.

**Figure 1:**
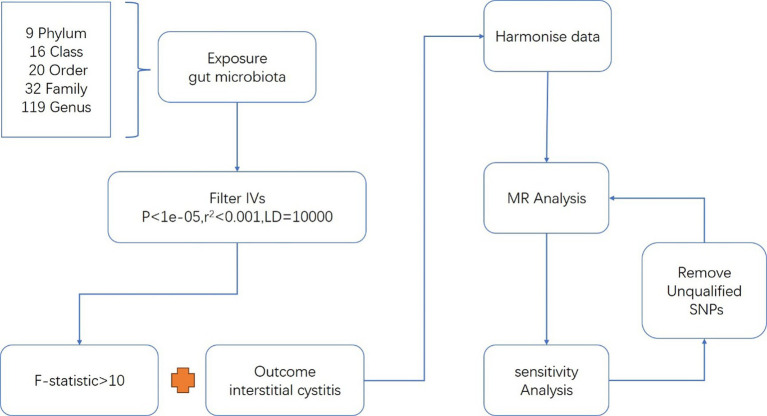
Workflow of the MR analysis.

Mendelian Randomization (MR) serves as a valuable tool for inferring causal relationships between exposure factors and disease outcomes through genetic variation (Sanderson et al., 2022). MR presents a convenient methodology for exploring potential protective and risk factors for disease, and has been implemented in diverse research investigations examining the relationship between gut microbiota and various diseases (Luo et al., 2022). The genome-wide association study (GWAS) summary datasets about gut microbiota and IC were applied to this analysis (Figure 1).

## 2 Methods

### 2.1 Study design

Our study methodology adhered to the STROBE-MR guidelines (Skrivankova et al., 2021; Supplementary material). Employing a two-sample Mendelian randomization (MR) design, we utilized summary-level statistics obtained from two distinct consortia. To mitigate potential bias, both exposure and outcome data were restricted to individuals of European ancestry. It is pertinent to highlight that all statistical data utilized in this investigation were sourced from publicly available databases, with participant consent and ethical approvals obtained, obviating the need for further permissions.

### 2.2 Instrumental variables (IVs)

Gut microbiota and IC were exposure factors and outcomes, respectively. Valid IVs must satisfy three key assumptions (de Leeuw et al., 2022): (1) The correlation hypothesis: IVs are strongly correlated with exposure. (2) The exclusivity hypothesis: IVs are independent of the outcome. (3) The independence assumption: IVs are independent of confounding factors. The criteria for selecting IVs were as follows: (1) Only independent SNPs associated with gut microbiota were considered, employing a clumping window size of 10,000 kb and an r2 threshold of less than 0.01 (Roze, 2023). (2) A significance threshold of *p*-value less than 1e-05 was applied to ensure an adequate number of SNPs for exposures (Sanna et al., 2019). (3) SNPs with F-statistics power below 10, indicative of potential weak instrumental bias, were excluded to mitigate this risk (Burgess and Thompson, 2011). (4) Additionally, palindromic SNPs were also excluded from consideration (Figure 1).

### 2.3 Data sources

Instrumental variables (IVs) for a total of 211 intestinal microflora (comprising 131 genera, 35 families, 20 orders, 16 classes, and 9 phyla) were obtained from a genome-wide meta-analysis conducted by the MiBioGen consortium (Wang et al., 2018; Kurilshikov et al., 2021). Further details of the study methodology have been described elsewhere (Kurilshikov et al., 2021). The study coordinated 16S rRNA gene sequencing profiles and genotyping data from a cohort of 18,340 participants across 24 cohorts, predominantly of European ancestry. Association analyses were performed with adjustments made for age, sex, technical covariates, and genetic principal components (Kurilshikov et al., 2021).

Summary statistics from GWAS on IC were acquired from GWAS catalog (Sollis et al., 2023),^1^ including 240 cases and 456,108 controls of European ancestry from UK Biobank. The UK Biobank, a cohort study encompassing 500,000 adults aged 40 to 69 years, was conducted across the UK from 2006 to 2010 (The Neale Lab, 2021). Diagnosis of IC relied on ICD 10-codes. Jiang and colleagues have developed an advanced genome-wide association (GWA) tool known as “fastGWA-GLMM,” specifically designed to handle large-scale GWAS datasets involving millions of individuals. This tool is capable of analyzing both common and rare variants across all binary phenotypes, even those characterized by highly imbalanced case–control ratios (Jiang et al., 2021). They have applied fastGWA-GLMM to investigate 2,989 binary traits using UK Biobank (UKB) data. The comprehensive summary statistics resulting from these analyses are publicly accessible through the fastGWA data portal.^2^

### 2.4 Statistical analysis

The F-statistic served as a crucial determinant for assessing the strength of IVs (Pierce et al., 2011):

R^2^ = 2 × MAF × (1 −MAF) × β^2^, F= R^2^ (n-k-1) / k(1-R^2^) “MAF” is the minor allele frequency of SNPs used as IVs, n means sample size, and k represents the number of SNPs.

A threshold F-statistic value of more than 10 indicated the absence of significant weak instrumental bias; otherwise, the respective instrumental variable was omitted from analysis. Various analytical methods, including Inverse Variance Weighted (IVW), MR Egger, Weighted Median, Simple Mode, and Weighted Mode, were employed to investigate the association between gut microbiota and IC. IVW was the primary method utilized (Burgess et al., 2019). Cochran’s Q tests were conducted to evaluate IV heterogeneity, with the random-effects IVW method selected in cases of significant heterogeneity (*p* < 0.05), while the fixed-effects IVW method was utilized otherwise (Greco et al., 2015). MR-Egger regression was employed to assess potential horizontal pleiotropy, with a *p*-value of the intercept less than 0.05 suggesting the presence of horizontal pleiotropy among SNPs (Burgess and Thompson, 2017). Additionally, the MR-PRESSO test was conducted to identify potential outliers among SNPs, with subsequent correction of association results by removing potential outliers (Verbanck et al., 2018). Leave-one-out analyses were conducted to identify potential IV heterogeneity.

## 3 Results

### 3.1 Main analysis

We screened 1,531 SNPs as instrumental variables from 196 gut microbiota. The F-statistics for these bacterial traits ranged from 14.58 to 88.42, all surpassing the threshold of 10, thereby mitigating concerns regarding weak instrument bias. Detailed Mendelian randomization (MR) analyses elucidating the associations between all 196 bacterial traits and the risk of IC are provided in Supplementary Table S2. Noteworthy findings, as discerned by the Inverse Variance Weighted (IVW) method, suggest the association of five bacterial traits with the risk of IC.

**Figure 2:**
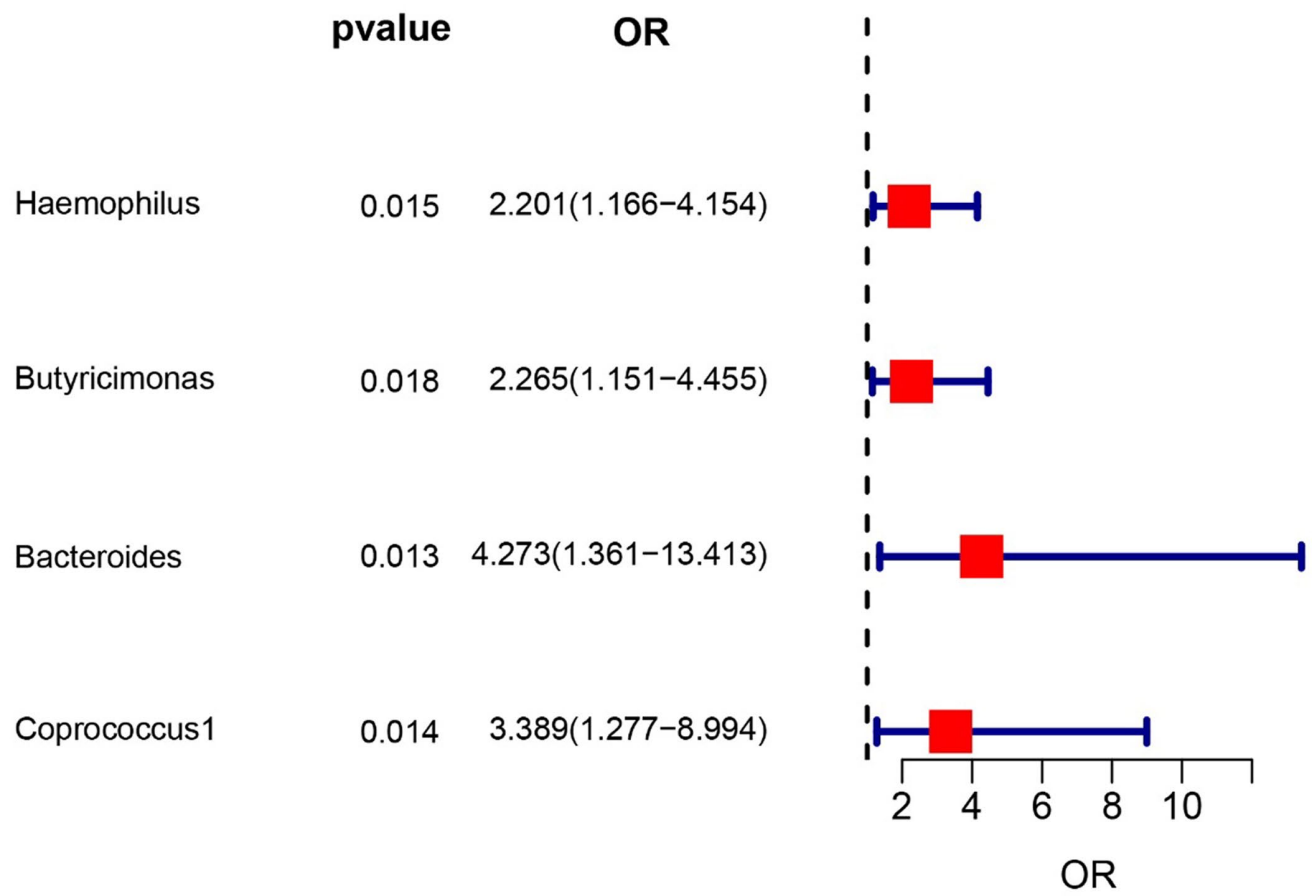
Forest plot of the associations between genetically determined 4 bacterial traits with the risk of interstitial cystitis.

To evaluate the potential impact of directional pleiotropy on the estimated causal effects, we conducted an assessment using the GWAS Catalog to identify single nucleotide polymorphisms (SNPs) associated with the five bacterial traits under investigation. Our scan revealed that only two SNPs (rs2013594 and rs946513) were found to be concurrently associated with other traits. These findings are detailed in Supplementary Table S4. After excluding the 2 SNPs, we recalculated the associations of the 5 genera with the risk of IC. Finally, 4 genera showing significant results for IVW analysis (Figure 2) were genus *Haemophilus* (OR = 2.20, 95% CI: 1.16–4.15, *p* = 0.015), genus *Butyricimonas* (OR = 2.26, 95% CI: 1.15–4.45, *p* = 0.018), genus *Bacteroides* (OR = 4.27, 95% CI: 1.36–13.4, *p* = 0.013) and *Coprococcus1* (OR = 3.39, 95% CI: 1.28–8.99, *p* = 0.014). The information of IVs used for these 4 bacterial traits are listed in Supplementary Table S1.

### 3.2 Sensitivity analysis

**Figure 3:**
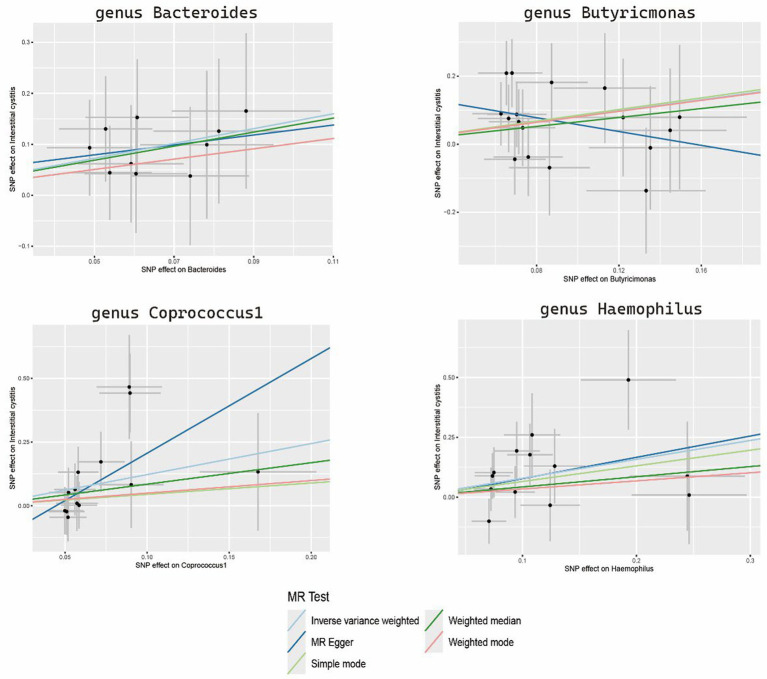
Scatter plots of the MR analysis.

The sensitivity analyses results are delineated in Table 1. Cochran’s Q-test outcomes did not yield significant values for any of the gut microbiota, indicating that none of the IVs were heterogeneous. Moreover, MR-PRESSO results indicated the absence of outliers. MR-Egger’s intercept analysis revealed no substantial evidence of horizontal pleiotropy, and directional estimates from each method were largely consistent, barring *Butyricimonas*. Notably, the MR-Egger method exhibited inconsistency in effect estimation for *Butyricimonas*, potentially attributable to its assumption of invalidity across all IVs, thereby compromising statistical power and precision (Figure 3). Consequently, its primary utility was for horizontal pleiotropy assessment. The leave-one-out technique demonstrated robustness in MR analysis outcomes, as illustrated in the Supplementary Figure S1, where removal of any SNP did not significantly alter results.

**Table 1:** Sensitivity analysis of significant gut microbiota.

Gut microbiota	Q_pval (IVW)	MR-PRESSO	MR-egger_test	
			Intercept	Pleiotropy test
*Haemophilus*	0.595	0.59	−0.012	0.895
*Butyricimonas*	0.767	0.796	0.159	0.147
*Bacteroides*	0.998	0.999	0.031	0.879
*Coprococcus1*	0.403	0.454	−0.166	0.149

## 4 Discussion

The gut microbiome exhibits associations with various human diseases. Clinical and animal model investigations have substantiated links between the gut microbiome, urinary microbiome, and UTIs (Magruder et al., 2019). Additionally, recent literature has increasingly acknowledged the relationship between intestinal flora and IC (Rahman-Enyart et al., 2021). However, observational studies fail to infer the precise direction of causality. Our study was the first to establish a causal relationship between gut microbiota and IC through Mendelian randomization (MR) analysis.

In this investigation, we utilized summary data from the largest genome-wide association study (GWAS) meta-analysis conducted by the MiBioGen Consortium on gut microbiota, along with summary statistics on IC derived from the GWAS Catalog, to explore potential causal links. Employing MR and sensitivity analyses, we examined filtered instrumental variables, revealing a causal relationship between specific gut microbiota and IC. Elevated levels of *Haemophilus*, *Butyricimonas*, *Bacteroides*, and *Coprococcus1* were associated with increased IC risk (OR > 1).

The association between these microbial flora and IC is a relatively novel finding in our study, with limited prior reporting. However, microbial strains linked to increased risk are frequently implicated in inflammation-related conditions. Notably, Bacteroides abundance exhibits significant sexual dimorphism, with higher levels observed in males across both IC and control groups (Zheng et al., 2023). Elevated levels of *Barnesiella* in the genitourinary system correlate with various urinary suppurative infections, encompassing acute and chronic urethritis, cystitis, and prostatitis (Brook, 2004). *Butyricimonas* species primarily produce butyric acid, with reduced production capacity reported in active ulcerative colitis cases; conversely, heightened *Butyricimonas* levels are observed in quiescent ulcerative colitis mucosa (Jangi et al., 2024). *Coprococcus1*, a member of the Lachnospiraceae family, is recognized as a key anaerobic bacterium in the intestinal milieu, exerting beneficial effects on sepsis-related 28-day mortality in critical care settings (Chen and Boyle, 2024). Additionally, a case–control study underscores differential urethral microbiota composition between men with and without idiopathic urethritis, with the genus *Haemophilus* implicated in the context of idiopathic urethritis (Plummer et al., 2022).

It is postulated that IC may be associated with dysregulation of the urine microbiome, potentially influenced by alterations in the intestinal microbiome. Siddiqui et al. examined the urinary microbiota of eight women diagnosed with IC for over four years, comparing their findings with data from healthy women in a previous study. Significant disparities between the urinary microbiota of IC patients and healthy counterparts were identified (Magruder et al., 2019). Magruder et al. contributed to our understanding of the gut microbiota-urinary tract infection axis, demonstrating an association between increased abundance of *Escherichia coli* (*E. coli*) in the gut and *E. coli*-induced UTI. Notably, gut *E. coli* strains exhibited a remarkable similarity to those detected in urine from the same subjects, supporting the notion of gut microbiota serving as a reservoir for urinary tract colonization and UTI (Antunes-Lopes et al., 2020). Furthermore, research has illuminated correlations between intestinal flora, IC, and inflammatory bowel disease (IBD). Next-generation sequencing advancements have unveiled dysbiosis—alterations in the composition and function of the gut microbiota—in IBD, with clinical and experimental evidence implicating dysbiosis in the pathogenesis of IBD (Nishida et al., 2018). Notably, patients with IBD often present with urinary disorders such as urinary urgency, increased frequency of urination, and difficulties in voiding (Jung et al., 2023). Moreover, the prevalence of IBD is markedly elevated in IC patients compared to healthy controls, indicating a strong association between these conditions (Alagiri et al., 1997).

One notable strength of our study lies in the application of MR analysis, which mitigates confounding and reverse causation biases often encountered in conventional observational studies. Consequently, our findings offer convincing evidence supporting the causal relationship between gut microbiota and IC. Furthermore, the utilization of data sourced from a sizable population enhances the statistical power of MR analysis, underscoring the robustness of our results.

This study is subject to several limitations. Firstly, our investigation was confined to a European population, potentially limiting the generalizability of our findings to other ethnic groups. Secondly, the taxonomic resolution was constrained to the genus level, precluding in-depth exploration of the causal relationship between gut microbiota and IC the species level. Additionally, the lack of subgroup information in the summary statistics for IC, such as categorization of IC, precluded the conduct of subgroup analyses.

## 5 Conclusion

Utilizing the power of Two Sample Mendelian randomization, we identified several microbiota types that play pivotal roles in enhancing the risk of IC. *Haemophilus*, *Butyricimonas*, *Bacteroides* and *Coprococcus1* were identified. These identified strains hold promise as prospective biomarkers and offer valuable insights into potential avenues for the treatment and prevention of IC. Moving forward, continued investigations are imperative to unravel the underlying mechanisms and clinical implications of these complex relationships.

## Data availability statement

The original contributions presented in the study are included in the article/Supplementary material, further inquiries can be directed to the corresponding author. The datasets presented in this study are deposited in NCBI Sequence Read Archive (SRA) and the accession number is PRJNA673102 (https://www.ncbi.nlm.nih.gov/bioproject/PRJNA673102/).

## Ethics statement

The requirement of ethical approval was waived by Ethics Committee of Huzhou First People’s Hospital for the studies on humans because all statistical data utilized in this investigation were sourced from publicly available databases, with participant consent and ethical approvals obtained, obviating the need for further permissions. The studies were conducted in accordance with the local legislation and institutional requirements. Written informed consent for participation was not required from the participants or the participants’ legal guardians/next of kin in accordance with the national legislation and institutional requirements. The human samples used in this study were acquired from gifted from another research group.

## Author contributions

JG: Writing – review & editing. SS: Writing – review & editing, Conceptualization. YS: Writing – original draft.

## Footnotes

^1^https://www.ebi.ac.uk/gwas/

^2^http://fastgwa.info/ukbimpbin
