# Supplementary figures and images for: Causal effects of gut microbiota on risk of interstitial cystitis: a two-sample Mendelian randomization study
Source: Front Microbiol. 2024 Jul 12;15:1434117. doi: 10.3389/fmicb.2024.1434117 (PMC11272578; doi:10.3389/fmicb.2024.1434117)

# MR Method

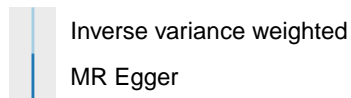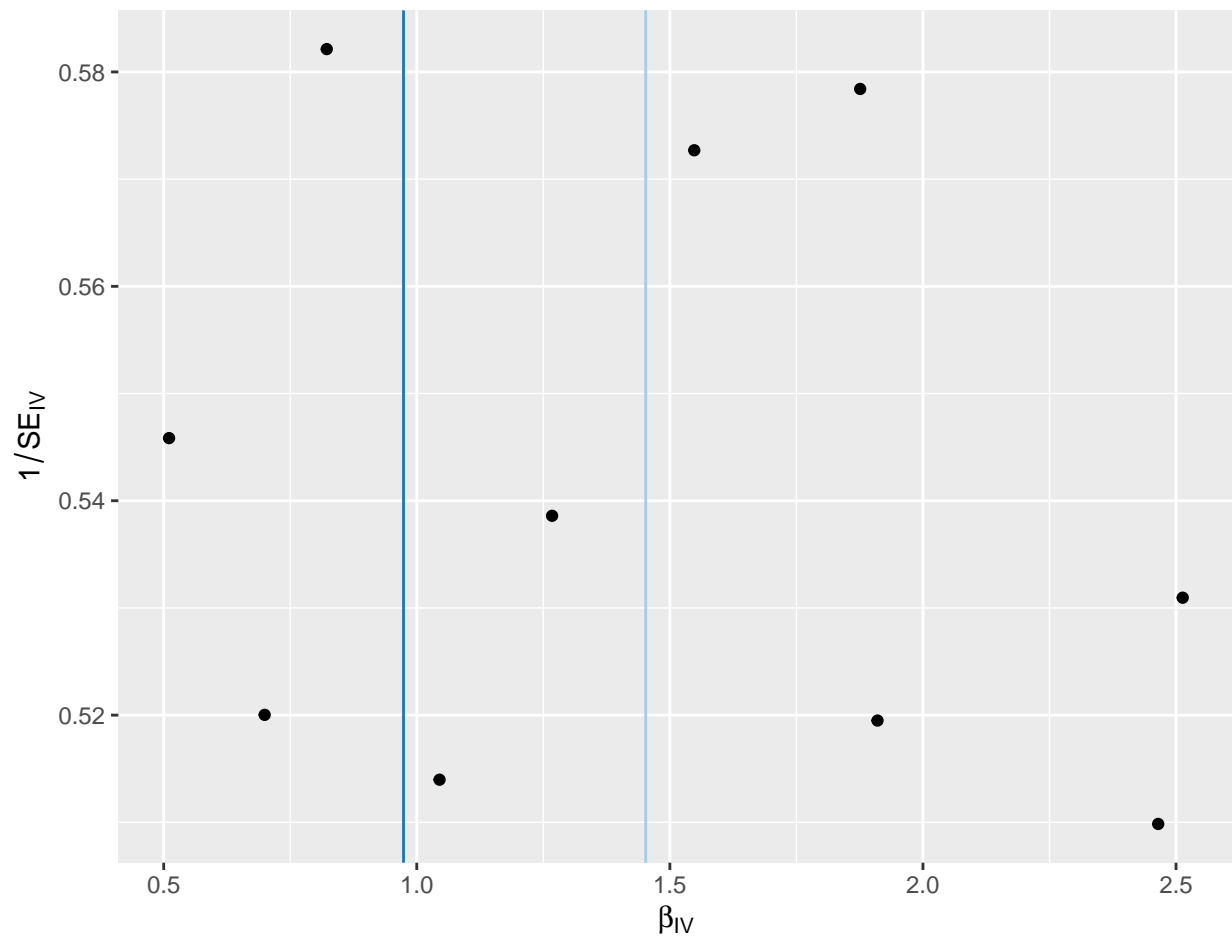

Supplement: Supplementary file 1 [file Data_Sheet_1.zip › New supplementary material/supplement figs/Bacteroides.funnel_plot.pdf]

# MR Method

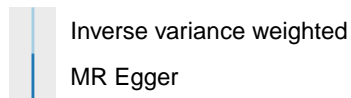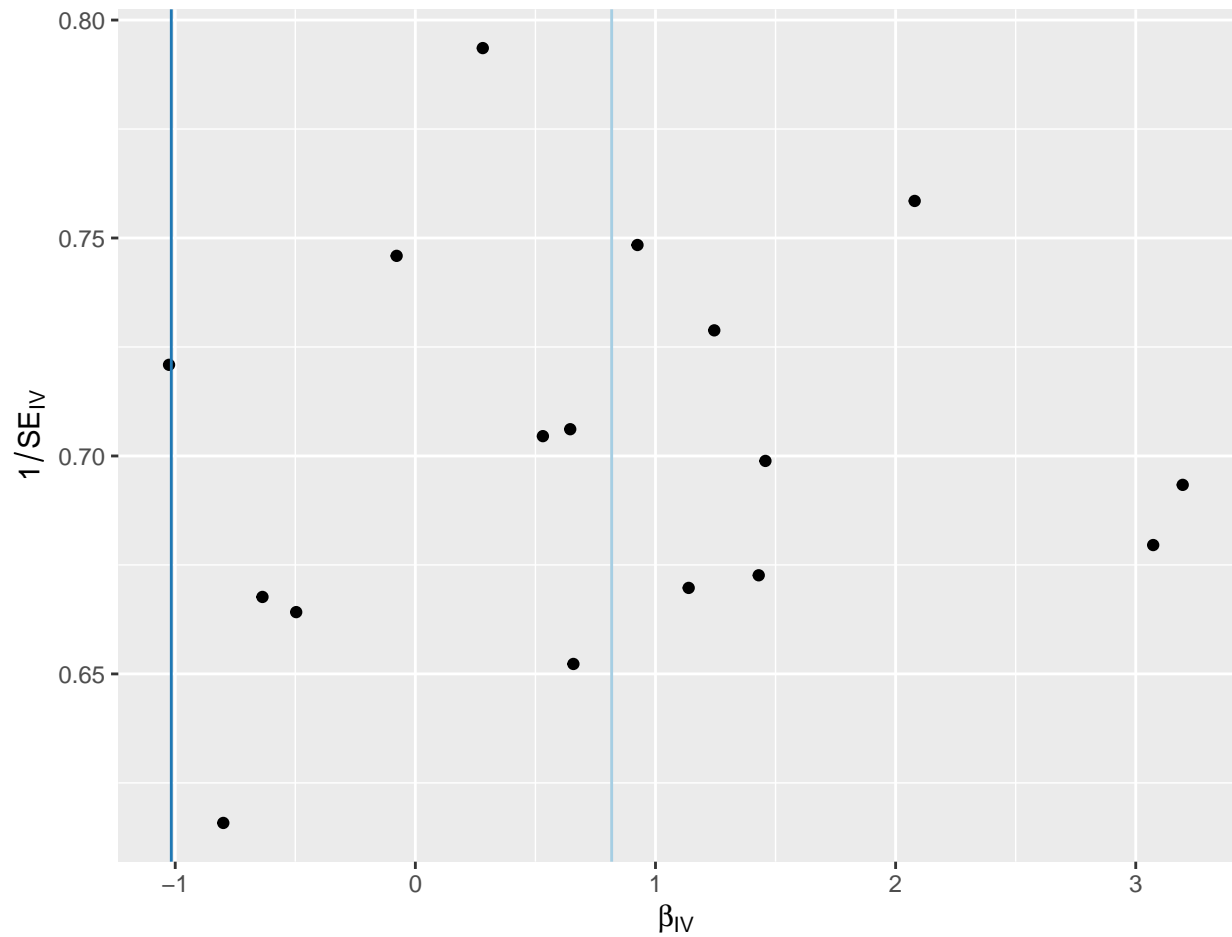

Supplement: Supplementary file 1 [file Data_Sheet_1.zip › New supplementary material/supplement figs/Butyricimonas.funnel_plot.pdf]

# MR Method

- Inverse variance weighted
- MR Egger

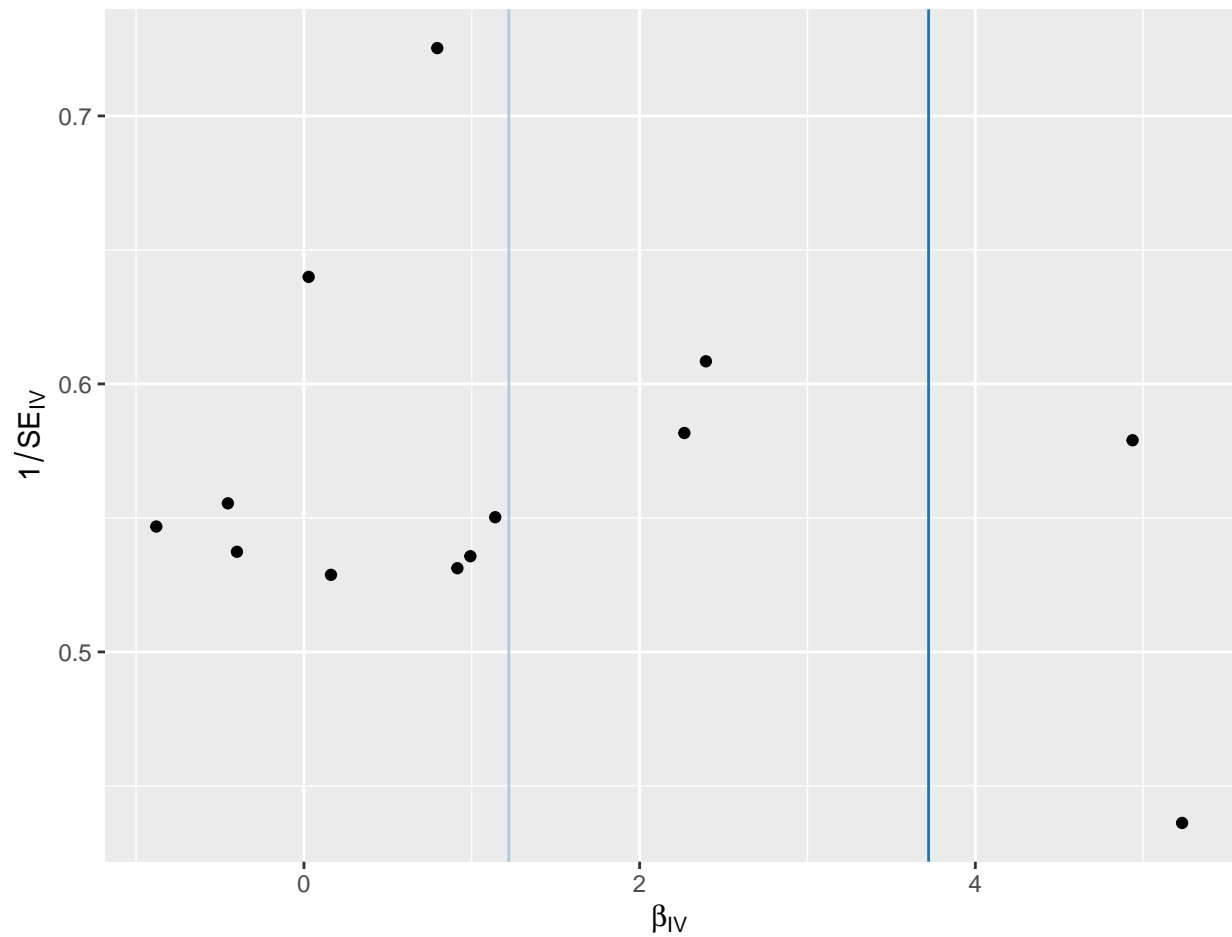

Supplement: Supplementary file 1 [file Data_Sheet_1.zip › New supplementary material/supplement figs/Coprococcus1.funnel_plot.pdf]

# MR Method

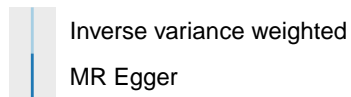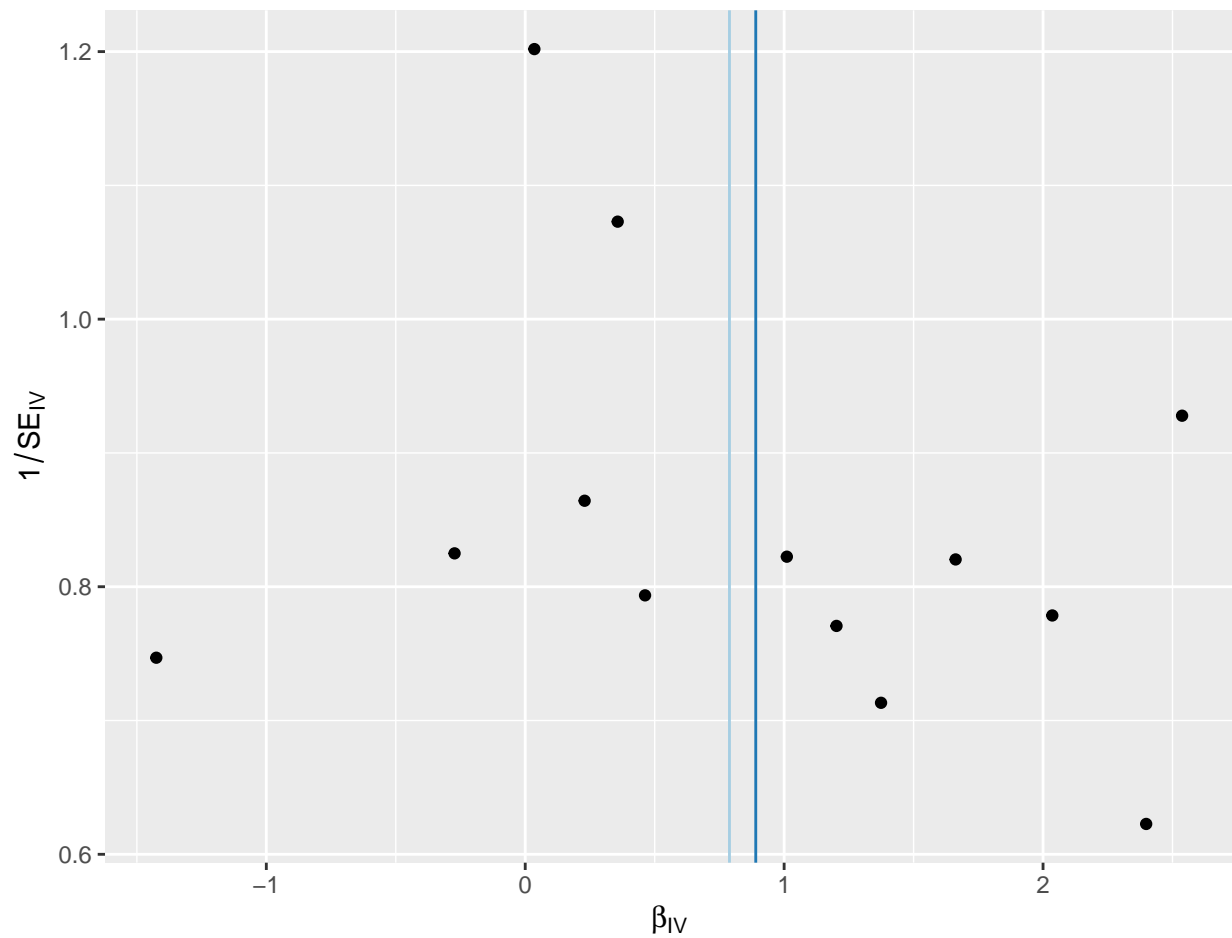

Supplement: Supplementary file 1 [file Data_Sheet_1.zip › New supplementary material/supplement figs/Haemophilus.funnel_plot.pdf]

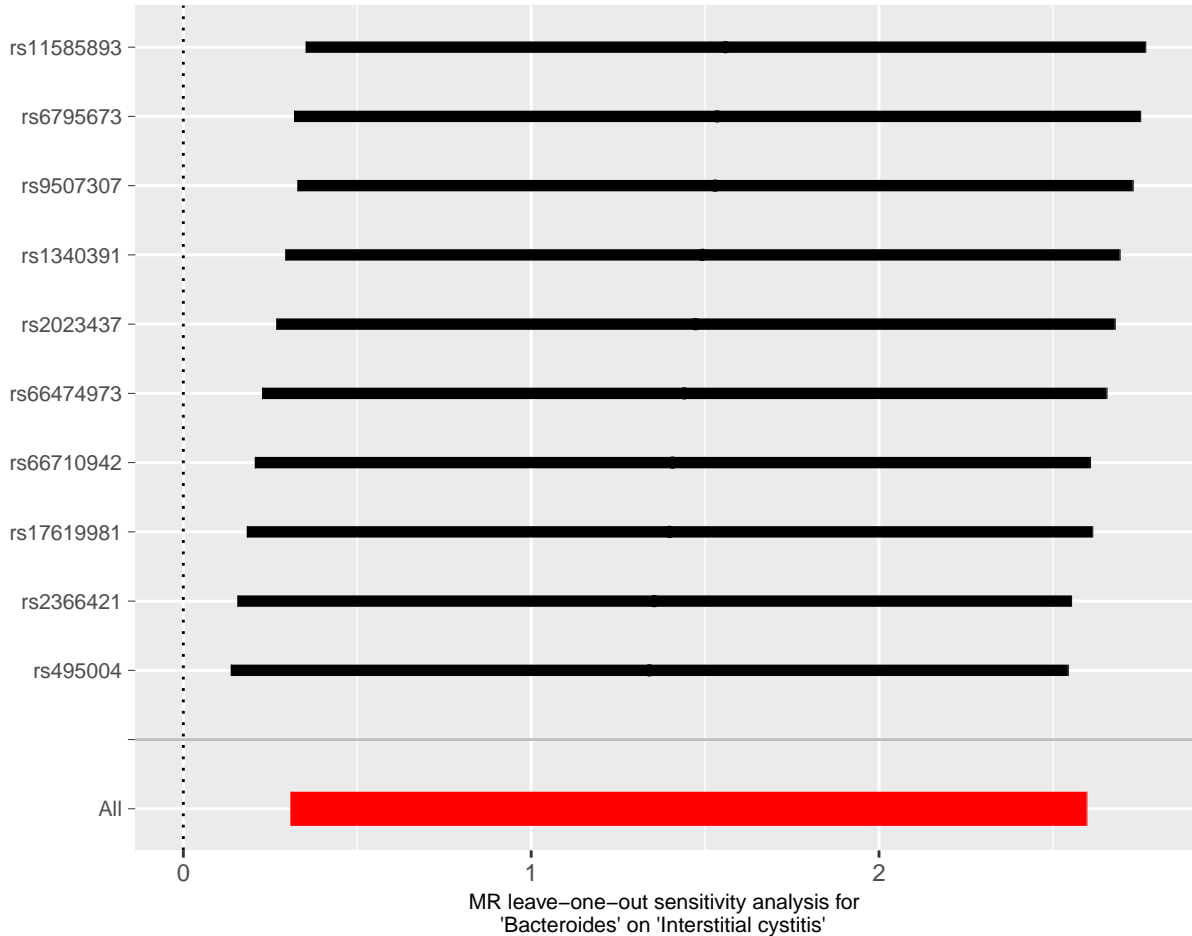

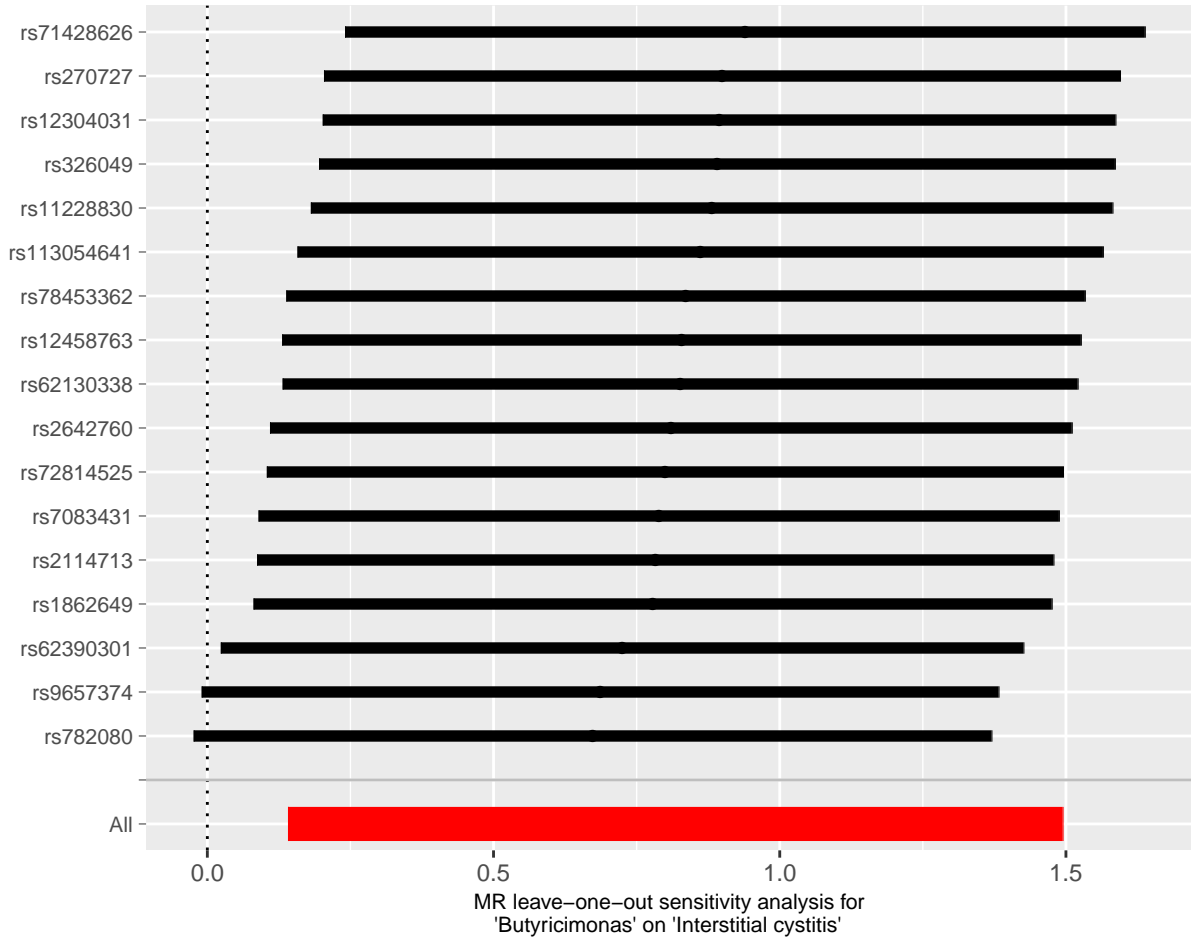

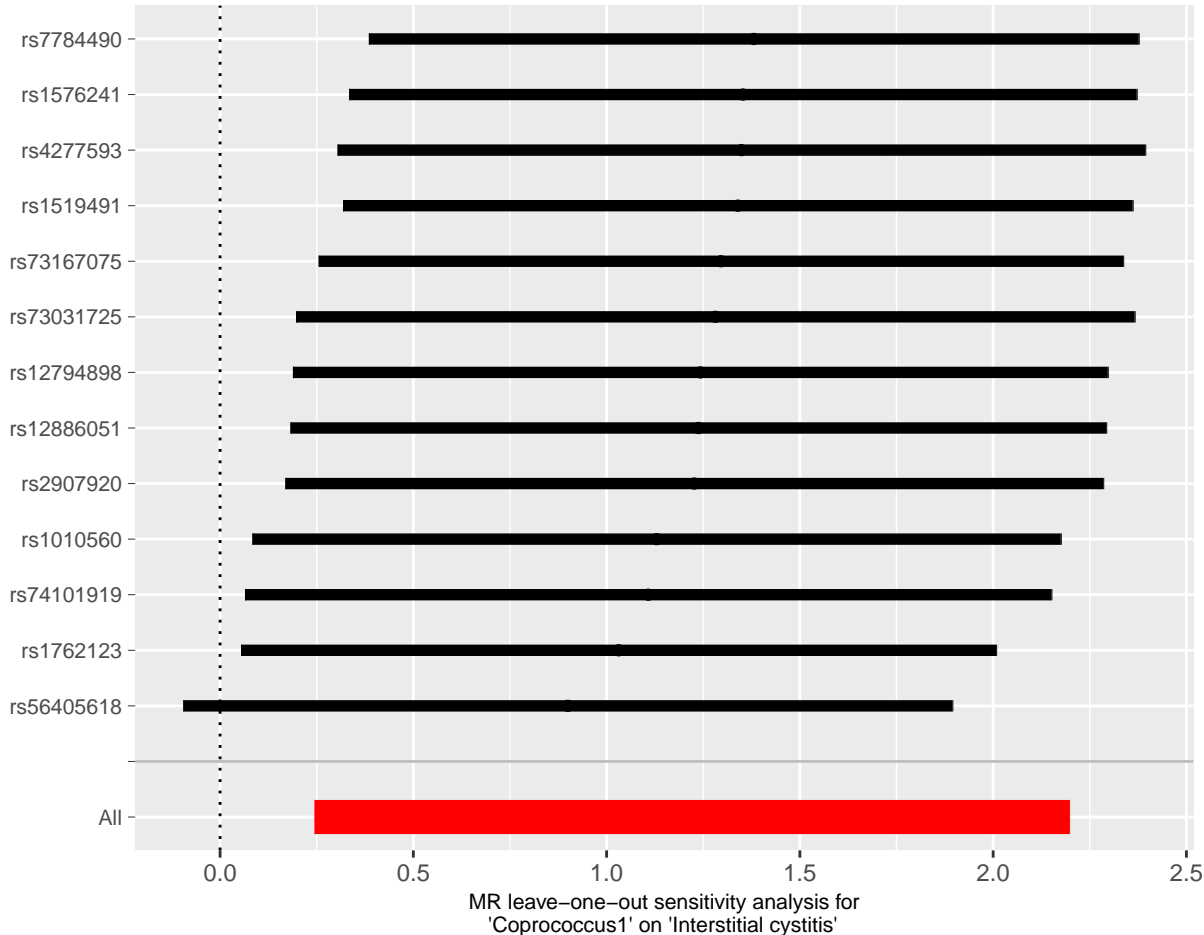

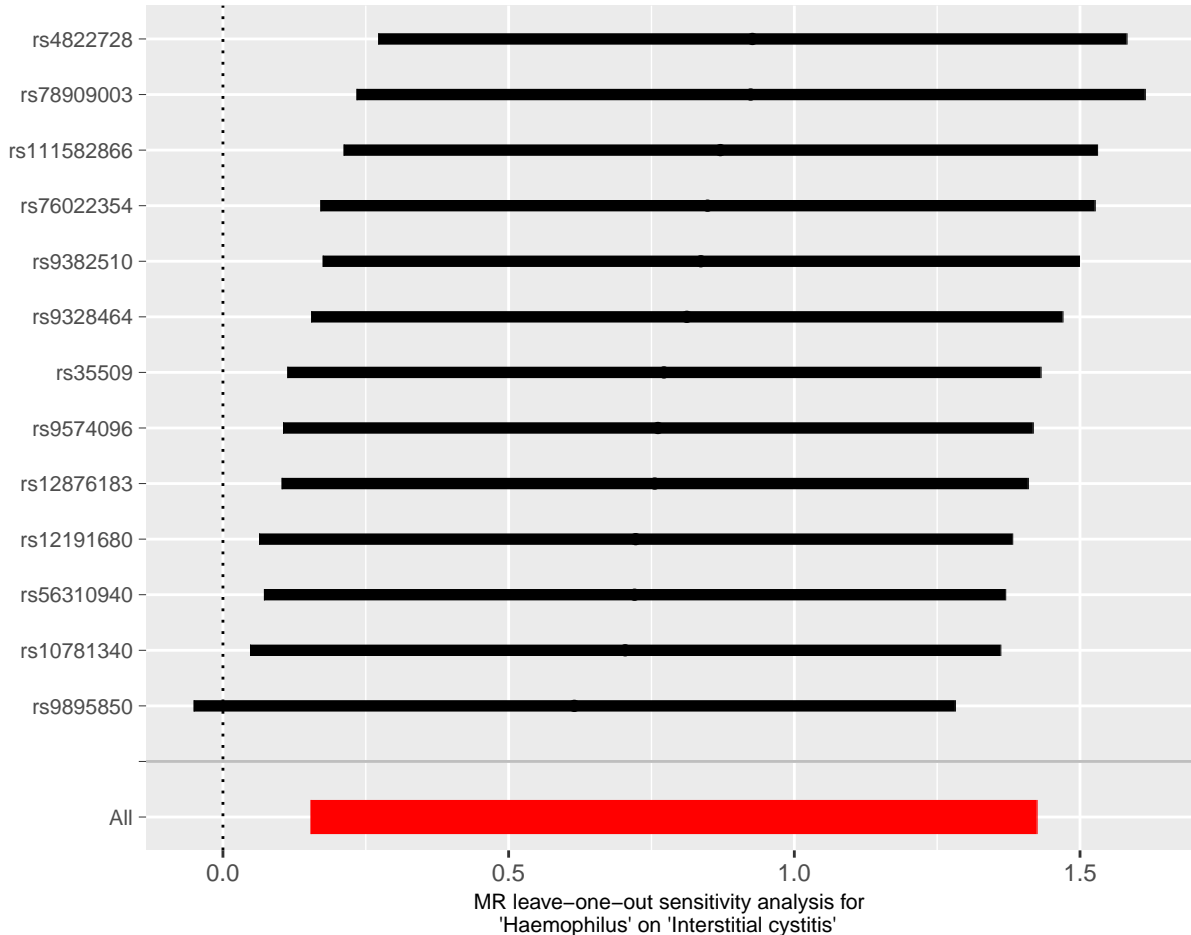

Supplement: Supplementary file 1 [file Data_Sheet_1.zip › New supplementary material/supplement figs/Supplementary Figure S1_MR leave_one_out sensitivity analysis.pdf]

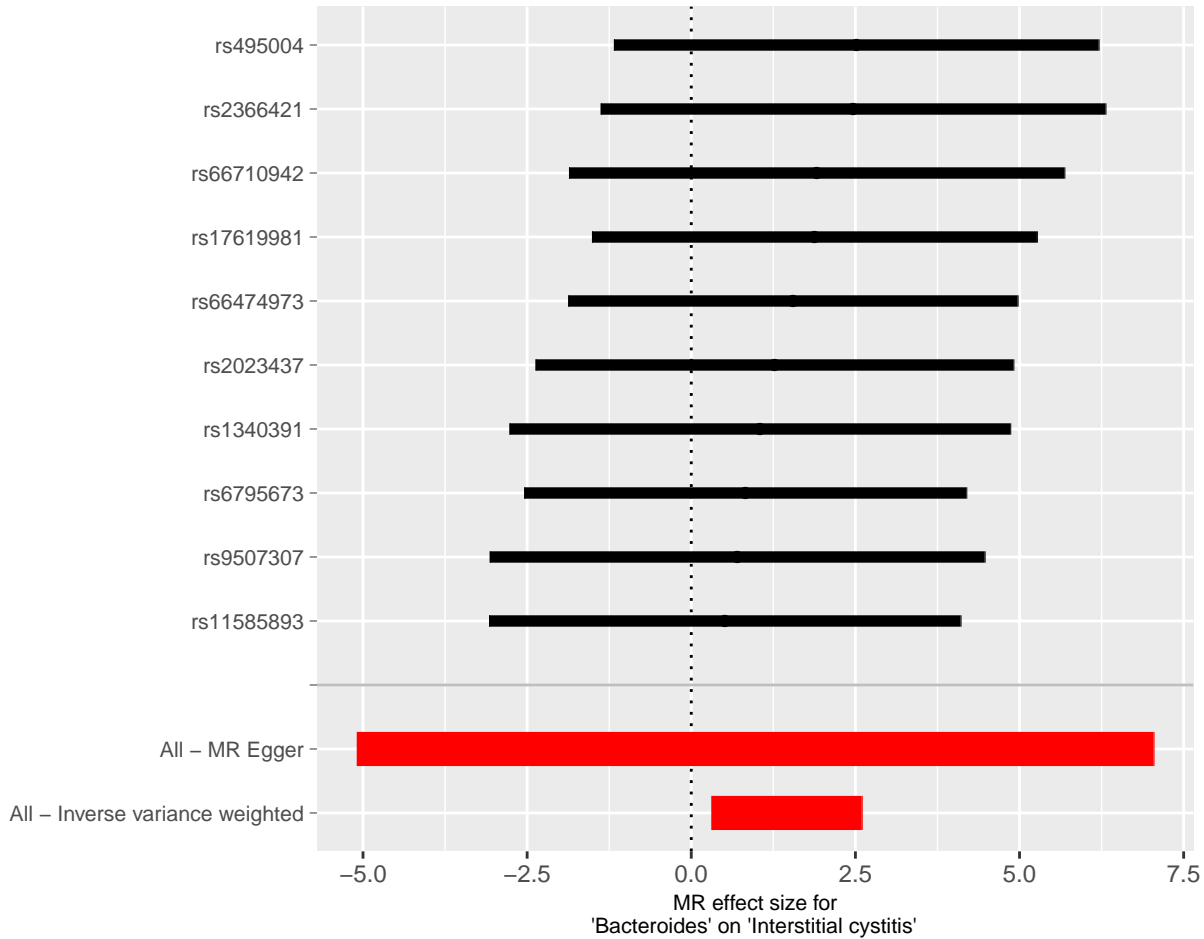

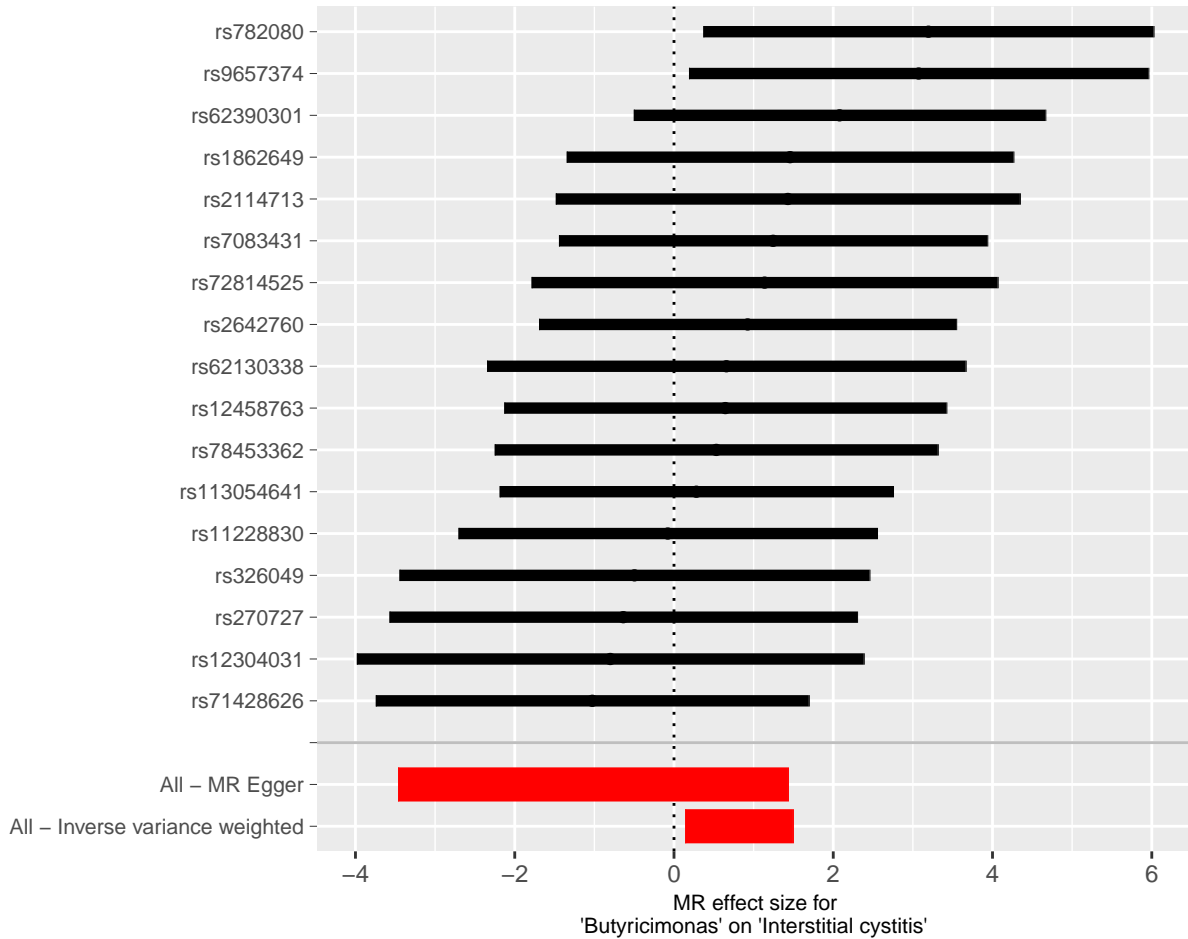

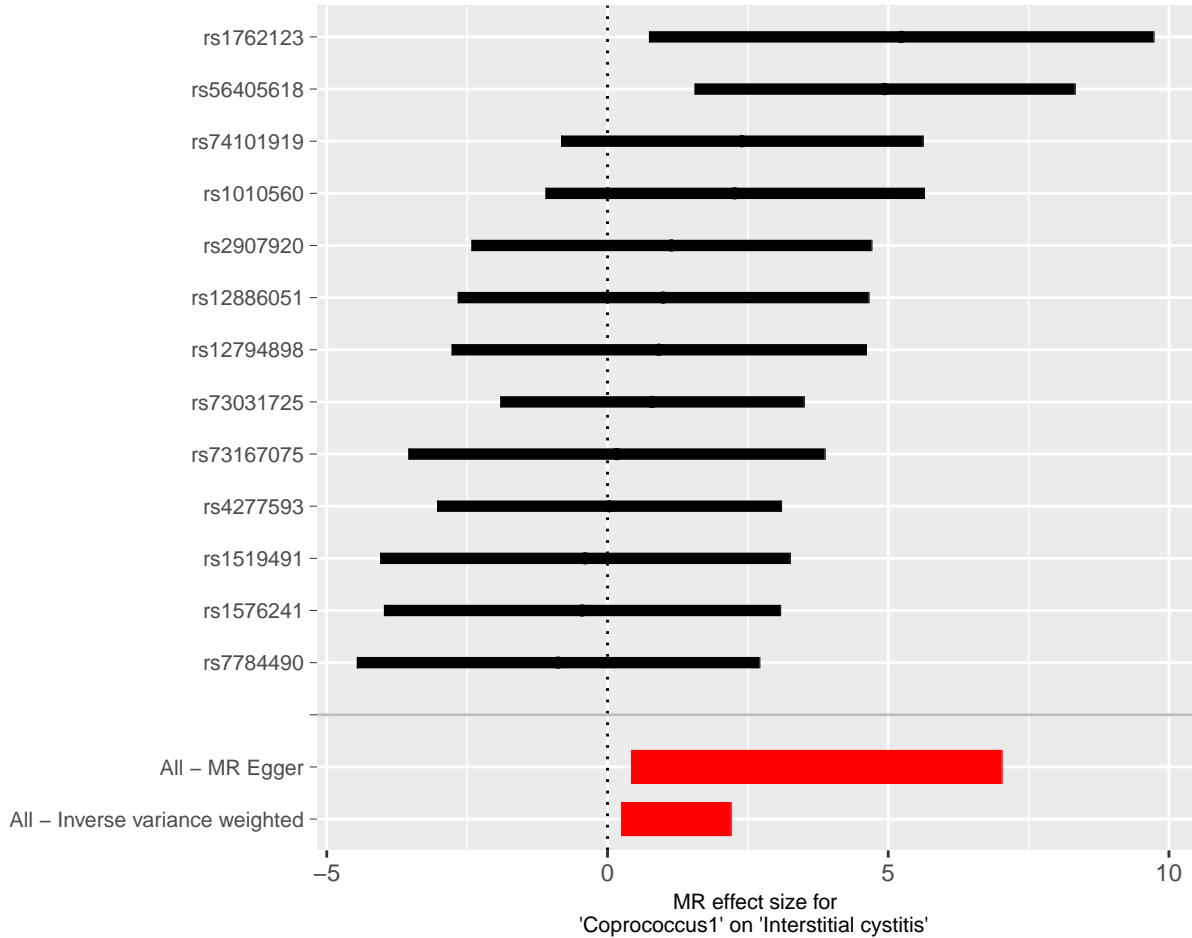

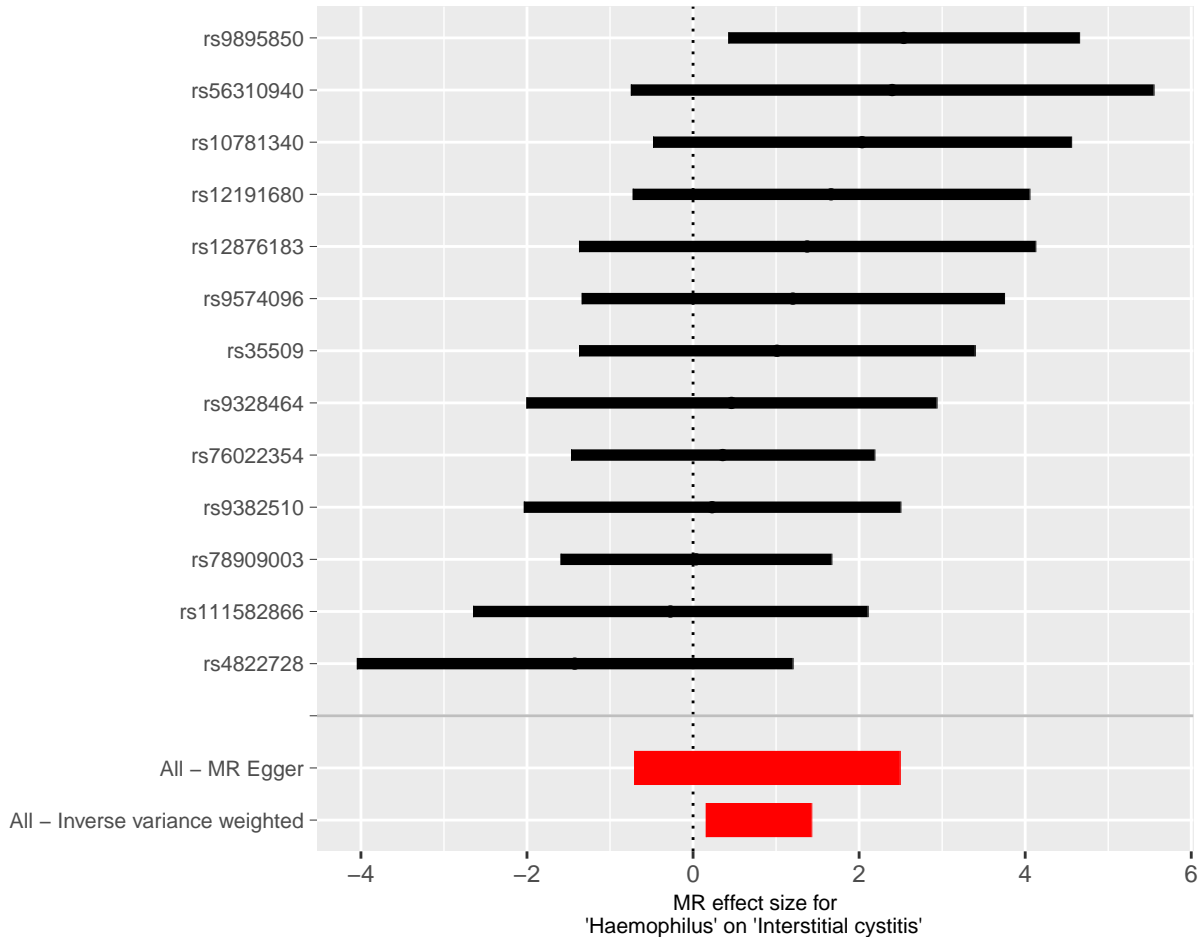

Supplement: Supplementary file 1 [file Data_Sheet_1.zip › New supplementary material/supplement figs/Supplementary Figure S2 single SNP forest.pdf]
